# Transcriptome Analysis Identifies Candidate Genes and Signaling Pathways Associated With Feed Efficiency in Xiayan Chicken

**DOI:** 10.3389/fgene.2021.607719

**Published:** 2021-03-17

**Authors:** Cong Xiao, Jixian Deng, Linghu Zeng, Tiantian Sun, Zhuliang Yang, Xiurong Yang

**Affiliations:** College of Animal Science and Technology, Guangxi University, Nanning, China

**Keywords:** RNA-seq, feed efficiency, candidate genes, protein–protein interaction, gene set enrichment analysis

## Abstract

Feed efficiency is an important economic factor in poultry production, and the rate of feed efficiency is generally evaluated using residual feed intake (RFI). The molecular regulatory mechanisms of RFI remain unknown. Therefore, the objective of this study was to identify candidate genes and signaling pathways related to RFI using RNA-sequencing for low RFI (LRFI) and high RFI (HRFI) in the Xiayan chicken, a native chicken of the Guangxi province. Chickens were divided into four groups based on FE and sex: LRFI and HRFI for males and females, respectively. We identified a total of 1,015 and 742 differentially expressed genes associated with RFI in males and females, respectively. The 32 and 7 Gene Ontology (GO) enrichment terms, respectively, identified in males and females chiefly involved carbohydrate, amino acid, and energy metabolism. Additionally, Kyoto Encyclopedia of Genes and Genomes (KEGG) analysis identified 11 and 5 significantly enriched signaling pathways, including those for nutrient metabolism, insulin signaling, and MAPK signaling, respectively. Protein–protein interaction (PPI) network analysis showed that the pathways involving *CAT*, *ACSL*1, *ECI*2, *ABCD*2, *ACOX*1, *PCK*1, *HSPA*2, and *HSP90AA*1 may have an effect on feed efficiency, and these genes are mainly involved in the biological processes of fat metabolism and heat stress. Gene set enrichment analysis indicated that the increased expression of genes in LRFI chickens was related to intestinal microvilli structure and function, and to the fat metabolism process in males. In females, the highly expressed set of genes in the LRFI group was primarily associated with nervous system and cell development. Our findings provide further insight into RFI regulation mechanisms in chickens.

## Introduction

Poultry is one of the healthiest meat sources due to its low fat and high protein content, and its health factors have led to an increase in its consumption in recent years (Liu et al., 2020). This increase in demand requires an increase in chicken feed on farms, leading to increased production costs. The cost of feed already makes up most of the total cost of poultry production (Aggrey et al., 2010). Therefore, improving feed efficiency plays an essential role in the production of poultry products.

Feed efficiency is generally estimated using residual feed intake (RFI), which was proposed in 1963 and is considered the most functional parameter for the evaluation of feed efficiency (Koch et al., 1963). At present, RFI has been applied in the artificial selection of feed efficiency in mammals and poultry (Mebratie et al., 2019; Banerjee et al., 2020; Liu and VandeHaar, 2020). There is a general agreement that RFI is a moderately inherited characteristic, making it easy to improve the feed efficiency of commercial breeding companies (Sell-Kubiak et al., 2017). The current RFI breeding process is mainly based on genomic selection and is expensive (VanRaden, 2020). However, the genes and biological processes that account for feed efficiency remain largely unknown. Therefore, there is an urgent need to develop effective biomarkers to facilitate RFI selection.

With the development of next-generation sequencing, it is possible to investigate the mechanisms underlying RFI and accelerate the breeding process of broiler chicken feed efficiency using molecular bioinformatics (Mardis, 2008; Shendure and Ji, 2008). High-throughput sequencing techniques have become powerful and effective tools for gaining a deeper understanding of the basic molecular mechanisms of complicated systems (Wang et al., 2009; Ozsolak and Milos, 2011). RNA sequencing, a high-throughput technique, has been generally used in livestock to find the expression patterns of functional genes (Cui et al., 2017; Ni et al., 2019; Peng et al., 2019).

In chickens, the duodenum is considered the main feed absorption organ. A previous duodenal transcriptome study indicated that RFI differences may be associated with digestion, metabolism, biosynthetic processes, and energy homeostasis (Yi et al., 2015). In addition, many studies have demonstrated that feed efficiency has a particular influence on mitochondrial function, intestinal cellularity and absorption, appetite regulation, and fat metabolism (Montanholi et al., 2013; Lancaster et al., 2014; Perkins et al., 2014; Reyer et al., 2017).

To date, abundant sequencing analyses have been carried out on commercial broiler breeds, but only a few studies have focused on indigenous chickens (Sun et al., 2013; Wolc et al., 2020). The physiological characteristics and genetic backgrounds of indigenous and commercial chicken breeds are quite distinctive. Thus, gaining insight into genetic resources is necessary for genetic studies of indigenous breeds. The Xiayan chicken is an indigenous chicken of the Guangxi province, in southern China. Due to its excellent meat quality, the Xiayan chicken is likely to become one of the preferred poultry breeds for consumers in the Guangxi and Guangdong provinces. Hence, this study was designed to identify the candidate genes and signaling pathways related to feed efficiency through the transcriptional sequencing of the duodenum in male and female Xiayan chickens. This will contribute to uncovering the molecular mechanisms of feed efficiency of indigenous chickens in Guangxi.

## Materials and Methods

### Ethics Statement

All animal experiments and methods in this study have been evaluated and approved by the Animal Ethics Committee of Guangxi University (GXU2018-058).

### Chicken Breed and RFI Calculation

According to the standard breed program, 340 indigenous Xiayan chicken (male = 173, female = 167) were bred in the Guangxi University experiment farm. The normal experiment started 70 to 90 days after the 10-day pre-experiment. The chickens were raised in 3-layer metal cages; the average stocking density was 15 birds per square meter. The basal diet was a corn-soybean broiler diet (13 MJ metabolizable energy/kg of diet, 220 g/kg crude protein) formulated without antibiotics or coccidiostats. *Ad libitum* access to fresh water was provided. Chickens were ranked by RFI, where the 10 most extreme duodenum samples from high (male = 3, female = 3) and low (male = 2, female = 2) RFI chickens were selected for RNA extraction. The feed intake (FI) and body weight (BW) were measured at 70–90 days of age. Feed conversion ratio (FCR) was calculated by FI and Body weight gain (BWG). The metabolic body weight (MBW^0.75^), BWG, and average daily body weight gain (ADG) were calculated according to the bodyweight of 70 and 90 days. ADFI was the average daily feed intake. The RFI value was used to measure the feed efficiency of Xiayan chickens, and we estimated it using the model as follows (Izadnia et al., 2019): RFI = ADFI-(b_0_ + b_1_MBW^0.75^ + b_2_ADG) where b_0_, b_1_, and b_2_ were regression intercept, the partial regression coefficient of ADFI on MBW^0.75^, and the partial regression coefficient of ADFI on ADG, respectively. SAS procedures *t*-test (SAS Version 9.4) was used to analyze the feed efficiency difference between HRFI and LRFI groups. The probability value was *P* < 0.05, indicating statistical significance. Detailed methods were described in our previous studies (Du et al., 2020).

### RNA Extraction and RNA Sequencing

Total RNA was extracted using the TRIzol^TM^ reagent (Invitrogen, Carlsbad, CA, United States) according to the manufacturer’s instructions. RNA purity and concentration were measured using the NanoPhotometer^®^ spectrophotometer (IMPLEN, Westlake Village, CA, United States) and Qubit^®^ RNA Assay Kit in Qubit^®^ 2.0 Fluorometer (Life Technologies, Carlsbad, CA, United States). RNA integrity was assessed using the RNA Nano 6000 Assay Kit of the Bioanalyzer 2100 system (Agilent Technologies, Santa Clara, CA, United States). Subsequently, sequencing libraries were generated using the rRNA-depleted RNA by NEBNext^®^ Ultra^TM^ Directional RNA Library Prep Kit for Illumina^®^ (NEB, United States) following manufacturer’s recommendations. Fragmentation was carried out using divalent cations under elevated temperature in NEBNext First Strand Synthesis Reaction Buffer (5X). First-strand cDNA was synthesized using random hexamer primer and M-MuLV Reverse Transcriptase (RNaseH-). Second-strand cDNA synthesis was subsequently performed using DNA Polymerase I and RNase H. In the reaction buffer, dNTPs with dTTP were replaced by dUTP. Remaining overhangs were converted into blunt ends via exonuclease/polymerase activities. After adenylation of 3′ ends of DNA fragments, NEBNext Adaptor with hairpin loop structure was ligated to prepare for hybridization. In order to select cDNA fragments of preferentially 150∼200 bp in length, the library fragments were purified with the AMPure XP system (Beckman Coulter, Beverly, United States). Then 3 μl USER Enzyme (NEB, United States) was used with size-selected, adaptor-ligated cDNA at 37°C for 15 min followed by 5 min at 95°C before PCR. Then PCR was performed with Phusion High-Fidelity DNA polymerase, Universal PCR primers, and Index (X) Primer. Then, products were purified (AMPure XP system) and library quality was assessed on the Agilent Bioanalyzer 2100 system. At last the libraries were sequenced on an Illumina Hiseq 4000 platform, and 150 bp paired-end reads were generated.

### RNA-Seq Data Analysis

Before read alignment, raw data (raw reads) in fastq format were first processed through Trimmomatic (Bolger et al., 2014). In this step, read bases with a phred base quality score less than 20, sequencing adapters, and reads containing poly-N are filtered to generate reliable clean data. Furthermore, Fastqc was used further to control the overall quality level of clean data to ensure the reliability of subsequent bioinformatics analysis (Schmieder and Edwards, 2011). The reference genome sequence files and annotation files used in this study were downloaded from the Ensembl genome browser^1^. Hisat2v2.1.0 was used to align clean data to a reference genome (Kim et al., 2015; Pertea et al., 2016). Afterward, stringtie (version 2.1.1) was used to assemble the transcriptome of each sample to generate a comprehensive transcript set (Pertea et al., 2015). With the fragments per kilobase of exon per million reads (FPKM) value, the gene expression levels can be quantified to a certain extent (Mortazavi et al., 2008). Furthermore, the assembly transcripts for all samples were integral to the enhancement of the overall quality of assembly by combining novel and mapped transcripts with a single one and removing the manual structures. The read count assignment was performed with the HTSeq-count tool of the HTSeq software (v0.6.1p1) (Anders et al., 2015). Prior to DEseq2 (version 2.2.1) (Love et al., 2014) read count standard normalization and expression analysis, genes with counts <1 were removed. Differently expressed genes (DEGs) were identified with | fold change| > 1 and *p*-value < 0.05. DEGs were picked out and analyzed in Kyoto Encyclopedia of Genes and Genomes (KEGG) and Gene Ontology (GO) by KOBAS software and Goseq in the R package. Benjamini-Hochberg (B-H) *p*-value < 0.05 was regarded as statistically significant.

### Protein–Protein Interaction (PPI) Network Construction and Modules Selection

Differently expressed genes were submitted to the online search tool STRING database^2^ to search for interacting genes to obtain gene interaction relationships, with a confidence score >0.9 defined as significant (Szklarczyk et al., 2019). The open-source software Cytoscape (Shannon et al., 2003) was used to visualize the PPI network of DEGs, and to visualize complex networks that provide a wide range of applications to analyze the interactive network further. The application of Molecular Complex Detection (MCODE) (Bader and Hogue, 2003) in Cytoscape was utilized to screen the modules of the PPI network. The standard settings of MCODE rest with: degree cutoff = 2, node score cutoff = 0.2, k-core = 2, maximum depth = 100. Additionally, the GO and KEGG enrichment analysis for the function and pathway of genes were carried out in the modules.

### Gene Set Enrichment Analysis (GSEA)

All expressed genes in both males and females could be applied in GSEA analysis by GSEA software, which ranked all expressed genes based on the fold-change (HRFI/LRFI) between the HRFI and LRFI groups (Bertocchi et al., 2019). The enrichment score of each gene set was then calculated with the full ranking, thus shedding light on the distribution of each gene set in the list. Furthermore, a normalized enriched score (NES) was determined for each gene set. The significant enrichment of the gene set followed the absolute values of NES > 1 and false discovery rate (FDR) ≤ 0.05 (Liu et al., 2018).

### Validation of RNA-Seq

Six DEGs were randomly selected. The primers used for qPCR were designed using Oligo 6.0 software. Five micrograms of RNA were reverse-transcribed into cDNA using RT Reagent Kit (TaKaRa, Dalian, China). The volume of the reaction mixture was 20 μl, with 2 μl of cDNA, 0.5 μl for each primer, 10 μl of SYBR (TaKaRa, Dalian, China), and 7 μl of RNA-free water. The following RT-PCR reaction was performed as follows: 95°C for 3 min; 95°C for 10 s, annealing temperature for the 30 s for 35 cycles; finally, melting curve collection at 65 to 95°C. The expression levels were calculated according to the 2^−ΔΔCt^ method normalized with β-actin. Primers used were synthesized by Sangon Biotech (Shanghai, China) and analyzed using Oligo 7.0 software. Primers for the samples *KMT2E*, *PPA*2, *IQGAP*2, *NPLOC*4, *SSH*1, and *TRAFD*1 genes are listed in Supplementary Table 1.

## Results

### Performance and Feed Efficiency

The disparity in ADFI, MBW0.75, ADG, RFI, and FCR is illustrated in Table 1. As expected, the FCR and RFI of the LRFI group were considerably lower than those of the HRFI group (*P* < 0.05), and the ADFI of the LRFI group accounted for approximately 80% of the HRFI group. In females, the ADFI and ADG of HRFI birds were significantly higher than those of LRFI birds (*P* < 0.05). Whereas in males, the RFI value of LRFI birds was −13.03 ± 0.92 compared with 13.56 ± 1.47 for the HRFI birds for 20 experimental days (day 70–90). In females, the LRFI birds had an RFI value of −11.79 ± 2.55 compared with 11.89 ± 1.62 for the HRFI birds (day 70–90). Moreover, there was no significant difference in metabolic body weight (MBW^0.75^) between the two groups (*p* > 0.05).

**TABLE 1:** Characterization of performance and feed efficiency traits of male and female (Least square means and SEM).

		HRFI	LRFI	*p*-value
Female	RFI, g/d	11.89 ± 1.62	−11.79 ± 2.55	< 0.001
	FCR, g:g	4.57 ± 0.09	3.0 ± 0.15	< 0.001
	ADFI, g/d	82.95 ± 1.69	65.63 ± 4.82	0.016
	MBW^0.75^, g	169.97 ± 5.90	178.38 ± 13.36	0.398
	ADG, g/d	18.17 ± 0.29	21.83 ± 0.95	0.015
Male	RFI, g/d	13.56 ± 1.47	−13.03 ± 0.92	< 0.001
	FCR, g:g	4.37 ± 0.20	2.75 ± 0.21	< 0.001
	ADFI, g/d	95.85 ± 6.73	75.30 ± 10.58	0.063
	MBW^0.75^, g	205.04 ± 14.54	203.15 ± 23.98	0.914
	ADG, g/d	22.0 ± 2.05	27.5 ± 4.58	0.162

*RFI, residual feed intake; FCR, feed conversion ratio; ADFI, average daily feed intake over the assessed feeding period; MBW^0.75^, mean of metabolic body weight; ADG, average daily gain over the assessed feeding period; HRFI, high residual feed intake; LRFI, low residual feed intake.*

### RNA-Seq Data

All duodenal samples of males and females (total *n* = 10) were gathered for RNA-seq. The amount of raw reads, clean reads, total mapped reads (%), uniquely mapped reads (%), Q20(%), Q30(%), and GC content (%) for each sample is shown in Supplementary Table 2. After the filter, the number of clean reads per sample ranged from 50,275,148 to 102,421,430. The general Q30 percentage of clean data was above 91%. Comparing the sequencing reads with the chicken reference genome, we found that the total map rate is between 81.92 and 91.79%, and the unique map rate is between 74.43 and 81.42%. The GC content of 10 samples ranged from 48.45% to 50.34%.

### Identification of DEGs

**FIGURE 1:**
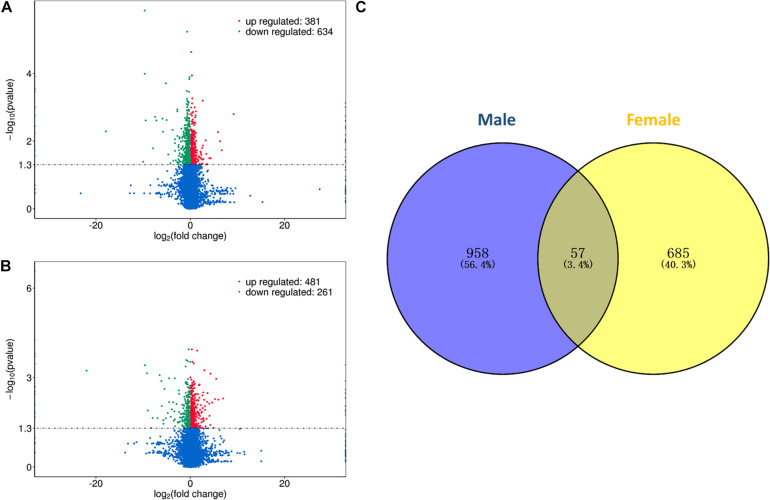
Differently expressed genes (DEGs) of transcriptome analysis. **(A,B)** Represent the volcano map of DEGs in the male and female, respectively. **(C)** Venn diagram showing the intersection of the DEGs between males and females.

In this study, differential expression analysis was performed to detect gene expression differences between the HRFI and LRFI groups. A total of 1015 and 742 genes were identified as being DEGs in males and females, respectively. Of the 1015 DEGs, 381 DEGs were upregulated in the HRFI groups, while 634 were downregulated than the LRFI groups (Figure 1A). In the females, 481 DEGs were upregulated in the HRFI groups, while 261 were downregulated compared with the LRFI groups (Figure 1B). 57 DEGs are shared between females and males (Figure 1C).

### GO and KEGG Analysis

**FIGURE 2:**
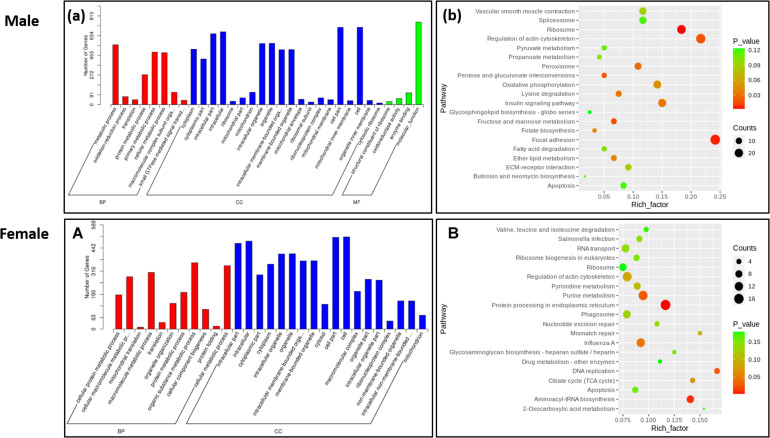
Function enrichment analysis of differently expressed genes. **(a)** GO enrichment analysis of DEGs in the Male. **(b)** KEGG enrichment analysis of DEGs in the male. **(A)** GO enrichment analysis of DEGs in the female. **(B)** KEGG enrichment analysis of DEGs in the female.

To further explore the functions of DEGs, we conducted a functional enrichment analysis. GO enrichment analysis showed that 8 and 11 GO items related to biological processes were significantly enriched in males and females, respectively. Notable among these were the metabolic process, cellular metabolic process, cellular metabolic process, and protein metabolic process. Other significant GO entries related to cellular components and molecular functions were oxidoreductase activity, mitochondrial part, enzyme binding cytoplasmic part, and membrane-bounded organelle (Figure 2).

Kyoto Encyclopedia of Genes and Genomes pathways enrichment analysis was performed to reveal the biological functions of DEGs further. In the male, 11 signaling pathways were significantly enriched, which mainly involved nutrient metabolism, energy metabolism, and insulin signaling pathways. Genes related to nutrient metabolism and energy metabolism were upregulated in the LRFI group, including *UQCRFS*1, *PCK*1, *ALDOB*, *EHHADH*, and *GCH*1. Simultaneously, genes involved in the insulin signaling pathway and calcium signaling pathway were upregulated in the HRFI group, including *VDAC*1, *PHKA*2, *PHKG*1, *PTGFR*, and *CACNA1C*. In the female, 5 pathways were significantly enriched, which were involved in endoplasmic reticulum protein processing and actin cells. The genes related to skeleton regulation and MAPK signaling pathway were upregulated in LRFI groups, while the genes involved in small molecule metabolism and synthesis were upregulated in the HRFI group.

### Identification of Hub Genes and Pathways Through PPI Network Analysis

**FIGURE 3:**
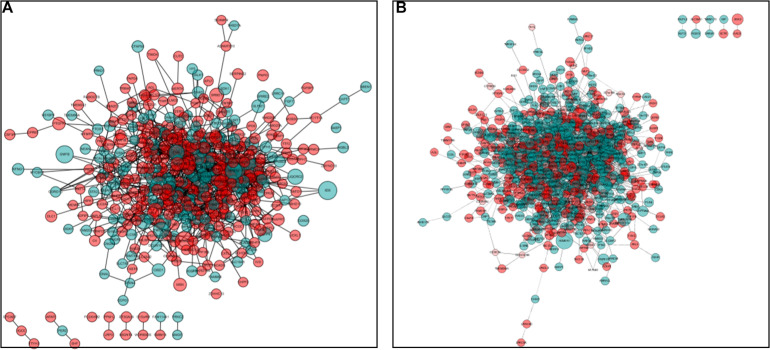
Protein–protein interaction (PPI) network analysis. **(A)** PPI network for DEGs in males. **(B)** PPI network for DEGs in the female. Red circles represent upregulated genes, and green circles represent downregulated genes. The size of the circle indicates the fold change of each gene (HRFI/LRFI).

**FIGURE 4:**
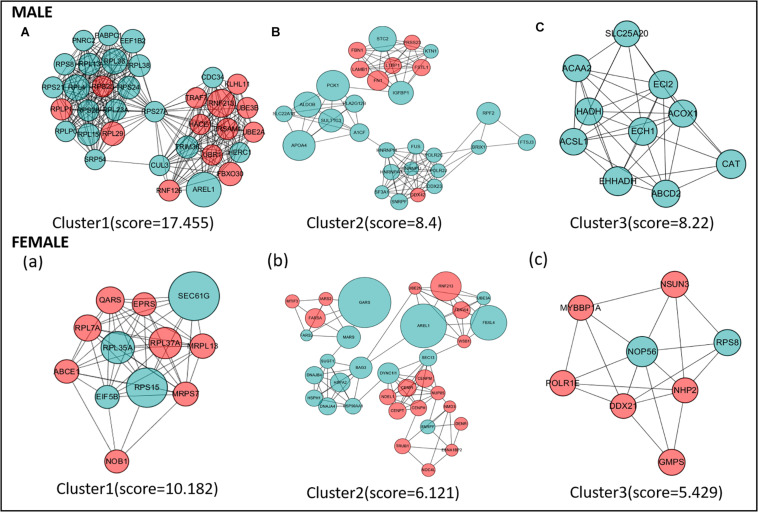
The top three protein–protein interaction (PPI) hub network modules in the male **(A–C)** and female **(a–c)**. Red circles represent upregulated genes, and green circles represent downregulated genes. The size of the circle indicates the fold change of each gene (HRFI/LRFI).

Protein–protein interaction network analysis was performed on DEGs; we could get more insight into the interaction relationship among them. The interaction relationship of DEGs was shown in Figure 3. We built the top three critical modules in DEGs’ PPI network through the MCODE application (Figure 4). And then, GO and KEGG enrichment analysis were performed on these genes in the modules. In the male, the top three modules’ genes were significantly enriched in the peptide biosynthetic process, peptide metabolic process, fatty acid degradation, peroxisome, fatty acid metabolism, and PPAR signaling pathway. In the female, these genes’ functions may be explained by the cellular response to heat, aminoacyl-tRNA biosynthesis, and ribosome. A complete result of the enrichment analysis of genes in each module was shown in Supplementary Table 3.

### GSEA

**FIGURE 5:**
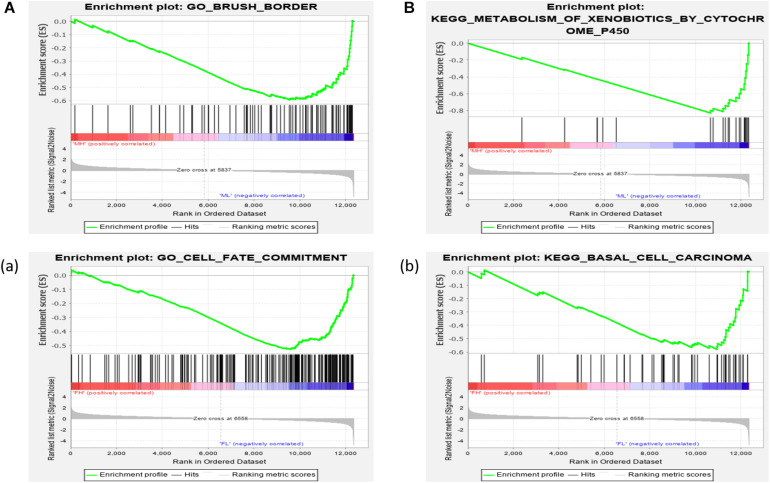
Gene set enrichment analysis (GO-base and KEGG-base). GSEA algorithm scored the enrichment of genes in the pathway in the ranked gene list. Enrichment score (ES) > 0 indicates that the distribution of the gene set is biased upstream of the ranking list, and ES < 0 shows the gene set distribution is biased downstream of the ranking list. **(A,B)**, **(a,b)** represent the GO entries and KEGG signaling pathways with the lowest FDR values. **(A)** Brush border and **(B)** Metabolism of xenobiotics by cytochrome p450 are enriched in the male LRFI groups. **(a)** Cell fate commitment and **(b)** Basal cell carcinoma are enriched in LRFI groups in the female.

We further investigated the difference in gene expression levels between HRFI and LRFI groups by GSEA. The results of the GSEA analysis were presented in Tables 2–5. As for the GO-based list, in the male, higher expression gene sets in the LRFI group were mainly associated with intestinal digestion and absorption, such as brush border, brush border membrane, and intestinal absorption. KEGG-base gene set enrichment analysis enriched primarily for xenobiotics’ metabolism by cytochrome p450, fatty acid metabolism, drug metabolism cytochrome p450, peroxisome, and PPAR signaling pathway. From the GO-based list, in the female, higher expression gene sets in the LRFI group were mainly connected to neurodevelopment, such as neuron fate commitment, central nervous system neuron differentiation, and neuron fate specification. The KEGG-base Gene set enriched primarily for basal cell carcinoma, hedgehog signaling pathway, neuroactive ligand-receptor interaction, and melanogenesis. The most enriched GO and KEGG items in the male and female were shown in Figure 5.

**TABLE 2:** Gene set enrichment analysis (GO-base) in the male.

GO-base list	NES	FDR	Higher expression group
Brush border	–2.26707	0.003079	LRFI
Peptide hormone processing	–2.25016	0.001539	LRFI
Brush border membrane	–2.24257	0.001026	LRFI
Intestinal absorption	–2.22644	0.001027	LRFI
Cluster of actin based cell projections	–2.168	0.00308	LRFI
Fatty acid metabolic process	–2.12511	0.004784	LRFI
Microbody lumen	–2.12404	0.004243	LRFI
Unsaturated fatty acid metabolic process	–2.10509	0.006005	LRFI
Cellular lipid catabolic process	–2.07536	0.009414	LRFI
Fatty acid biosynthetic process	–2.06612	0.009701	LRFI
Extracellular matrix structural constituent conferring tensile strength	2.297024	0.001245	HRFI
Extracellular matrix structural constituent	2.187153	0.004879	HRFI
Basement membrane	2.186745	0.003253	HRFI
Collagen fibril organization	2.172451	0.004158	HRFI
Collagen trimer	2.080334	0.019748	HRFI
Neuron projection extension involved in neuron projection guidance	1.992951	0.060019	HRFI
rRNA methylation	1.986123	0.05773	HRFI
Complex of collagen trimers	1.98479	0.051003	HRFI
Cartilage morphogenesis	1.965841	0.057518	HRFI
Catalytic activity acting on a rRNA	1.965179	0.052458	HRFI

*NES, enrichment score; FDR, false discovery rate of the *p*-value.*

**TABLE 3:** Gene set enrichment analysis (KEGG-base) in the male.

KEGG set	NES	FDR	Higher expression in HRFI or LRFI
Metabolism of xenobiotics by cytochrome p450	–2.40002	5.02E-06	LRFI
Fatty acid metabolism	–2.15454	6.10E-04	LRFI
Drug metabolism cytochrome p450	–2.13537	4.07E-04	LRFI
Peroxisome	–2.00773	0.002549	LRFI
PPAR signaling pathway	–1.93454	0.0089	LRFI
Glutathione metabolism	–1.86585	0.016234	LRFI
Oxidative phosphorylation	–1.86476	0.014085	LRFI
Pyruvate metabolism	–1.85897	0.012981	LRFI
Retinol metabolism	–1.84681	0.01302	LRFI
Drug metabolism other enzymes	–1.79034	0.021592	LRFI
Focal adhesion	1.906565	0.033723	HRFI
ECM receptor interaction	1.828651	0.036793	HRFI

*NES, enrichment score; FDR, false discovery rate of the *p*-value.*

**TABLE 4:** Gene set enrichment analysis (GO-base) in the female.

GO-base list (CC, BP, MF)	NES	FDR	Higher expression in HRFI or LRFI
Cell fate commitment	–2.43399	4.52E-06	LRFI
Neuron fate commitment	–2.42067	6.07E-05	LRFI
Mesonephros development	–2.34885	0.000405	LRFI
Golgi lumen	–2.31769	0.000612	LRFI
Central nervous system neuron differentiation	–2.30507	0.000741	LRFI
Cell differentiation in spinal cord	–2.27065	0.001833	LRFI
Diencephalon development	–2.26932	0.001571	LRFI
Cell fate specification	–2.26658	0.001525	LRFI
Cardiocyte differentiation	–2.2519	0.001756	LRFI
Neuron fate specification	–2.24478	0.00158	LRFI
Centromere complex assembly	2.479677	1.56E-05	HRFI
Chromatin remodeling at centromere	2.461585	3.87E-05	HRFI
DNA packaging	2.36867	0.000282	HRFI
DNA replication independent Nucleosome organization	2.361665	0.000212	HRFI
Histone exchange	2.32466	0.000169	HRFI
DNA conformation change	2.32324	0.000141	HRFI
Chromatin assembly	2.248558	0.000244	HRFI
ATP dependent chromatin remodeling	2.241777	0.000214	HRFI
Kinetochore organization	2.207554	0.000381	HRFI
Interferon gamma mediated signaling pathway	2.196199	0.000343	HRFI

*NES, enrichment score; FDR, false discovery rate of the *p*-value.*

**TABLE 5:** Gene set enrichment analysis (KEGG-base) in the female.

KEGG set	NES	FDR	Higher expression in HRFI or LRFI
Basal cell carcinoma	–2.11055	0.003972	LRFI
Hedgehog signaling pathway	–2.0326	0.003453	LRFI
Neuroactive ligand receptor interaction	–1.84756	0.026776	LRFI
Melanogenesis	–1.8143	0.026804	LRFI
Gap junction	–1.79889	0.023586	LRFI
DNA replication	2.19206	0.001313	HRFI
Primary immunodeficiency	2.084619	0.002965	HRFI
Mismatch repair	2.020003	0.003884	HRFI
Intestinal immune network for IgA production	1.974355	0.004076	HRFI
Cell cycle	1.955905	0.004391	HRFI
B cell receptor signaling pathway	1.7872	0.029919	HRFI
Autoimmune thyroid disease	1.783557	0.027321	HRFI
Pyrimidine metabolism	1.727826	0.045972	HRFI
Porphyrin and chlorophyll metabolism	1.718103	0.04515	HRFI
One carbon pool by folate	1.698381	0.049032	HRFI

*NES, enrichment score; FDR, false discovery rate of the *p*-value.*

### Validation of RNA-Seq

**FIGURE 6:**
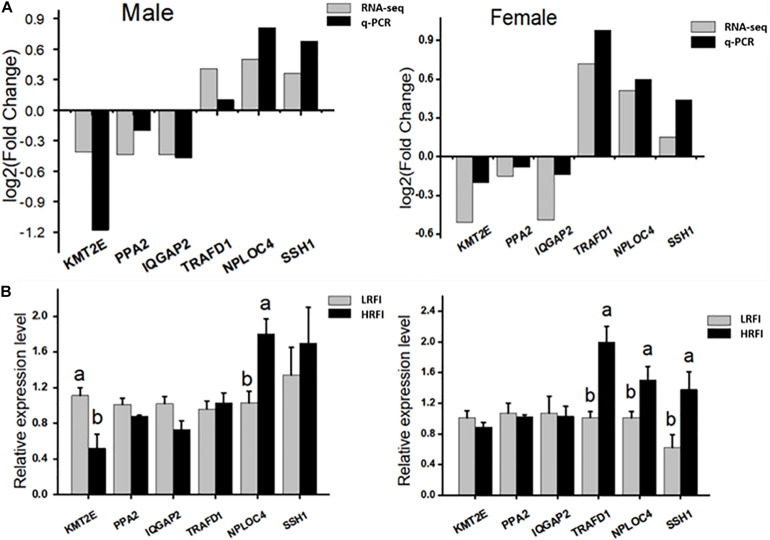
Validation of the accuracy of RNA-seq. **(A)** Correlations of the expression level of 6 random DEGs between high and low abdominal fat using RNA-Seq and qPCR. The *x*- and *y*-axis correspond to the genes and log2 (ratio of H/L) measured by RNA-Seq and qPCR. **(B)** Gene expression abundance of HRFI and LRFI in Male and female; results are expressed as means ± standard deviation (*n* = 6); a, b means *p* < 0.05.

Six genes were selected randomly from DEGs for qPCR validation. The results showed that *KMT2E*, *PPA*2, and *IQGAP*2 were upregulated in the LRFI group, while *TRAFD*1, *NPLOC*4, and *SSH*1 were upregulated in the HRFI group. The expression patterns of six genes in qPCR were consistent with those in RNA-seq (Figure 6).

## Discussion

Feed efficiency plays an important role in improving profits and the environmental footprint in broiler production. In this study, the duodenum transcriptome data came from four groups of Xiayan chickens with extreme opposing RFI and different sex using RNA-seq. The gene expression profile was further explored by differential expression analysis, GO and KEGG enrichment analysis, PPI network analysis, and GSEA. All bioinformatics analyses were conducted to study gene expression differences, associations, and enrichment to further gain more widespread biological insight into indigenous chickens’ feed efficiency.

Among males and females, the values of RFI, FI, and FCR of high-RFI chickens were significantly higher than those of low-RFI chickens. However, there was no significant difference between ADG, BW, and MBW, which was consistent with the findings of previous studies (Zhang X. et al., 2017; Zhang et al., 2019). RFI uses feed intake to measure feed efficiency; using RFI to select feed efficiency in breeding work can avoid affecting other growth traits. RFI could be used as an ideal breeding index for chicken feed efficiency trait.

Traditional differential expression analysis of RNA sequencing data would produce many DEGs, and further analysis was needed to understand the function of the DEGs between different comparison groups (Hekman et al., 2015). Therefore, we further performed functional enrichment analysis on these DEGs. The analysis results of GO and KEGG showed that these genes were mainly involved in metabolism progress, including material metabolism processes, protein metabolism, carbohydrate metabolism, amino acid metabolism, energy metabolism, and lipid metabolism processes. Many studies have shown that metabolism is an essential factor affecting residual feed intake (Jing et al., 2015; Yi et al., 2015; Kong et al., 2016).

In males, some genes related to the metabolism process were upregulated in the LRFI group, such as *ALDOB* (Aldolase, Fructose-Bisphosphate B), *UQCRFS*1 (ubiquinone-cytochrome C reductase iron-sulfur protein subunit Base 1), *EHHADH* (3-hydroxyacyl-CoA dehydrogenase), and *GCH*1 (guanosine triphosphate cyclase I). A notable gene is *ALDOB*. The aldolase family is a biological enzyme necessary for bioenergy metabolism. It catalyzes the formation of dihydroxyacetone phosphate and glyceraldehyde 3-phosphate by fructose 1, 6-bisphosphate, which plays a vital role in glycolysis (Almon et al., 2007). *ALDOB* (aldolase B), as a member of the aldolase family, is mainly distributed in liver tissues, participates in liver metabolism, and also participates in glycolysis and gluconeogenesis processes (Kallio et al., 1998). More recent studies already point to *ALDOB* as a candidate gene for feed efficiency in chickens. A previous study in chicken found the *ALDOB* gene that participates in glycolysis and gluconeogenesis was highly expressed in LRFI chickens (Shah et al., 2019). Gene expression in breast muscle associated with feed efficiency in a single male broiler line using a chicken 44K oligo microarray also identified that *ALDOB* was more highly expressed in high feed efficiency chickens (Kong et al., 2011). We speculate that *ALDOB* could have an essential role in enhancing energy metabolism in the high-FE broiler phenotype. Some genes enriched in the insulin signaling pathway were upregulated in the HRFI group. In the chicken, the insulin-signaling pathway has anabolic effects in glucose transport and utilization, glycogen synthesis, control of liver lipogenic enzymes, amino acid transport, and protein synthesis (Fujita et al., 2019). Previous genome-wide association analysis identified an SNP site significantly related to feed efficiency that may regulate feed efficiency through the insulin signaling pathway (Yuan et al., 2017). A study conducted on Duroc pigs with differences in RFI has found that the insulin signaling pathway may be a biological process that is significantly associated with RFI (Banerjee et al., 2020). Our findings were consistent with the results above, indicating the body’s digestion and metabolism are essential to RFI, and high feed efficiency chickens may have a stronger ability to metabolize nutrients. In the female, genes related to the MAPK signaling pathway and actin cytoskeleton regulation were upregulated in the LRFI group. Many studies have proven that MAPK plays a key role in the regulation of energy balance (Kikuchi et al., 2020; Okubo et al., 2020). Previous studies have shown that the MAPK signaling pathway is widely involved in the regulation of growth and development (Xu et al., 2019; Zhao et al., 2019); the genes contained in the MAPK signaling pathway may regulate feed efficiency through energy distribution and homeostasis in the digestion, absorption, and metabolism of feed.

Protein–protein interaction network analysis can capture the interaction information of DEGs, which helps to understand the molecular mechanism of fat deposition from the perspective of biological systems (Athanasios et al., 2017). In this research, the PPI network analysis was constructed with DEGs. To identify the essential part of the PPI network, we used the “module” program to extract the top three modules and performed GO and KEGG enrichment analysis. Some GO items and KEGG signaling pathways were worth noting, including fatty acid degradation, fatty acid metabolism, PPAR signaling pathway, and cellular response to heat. Some biological pathways, like lipid metabolism and cholesterol biosynthesis, were identified to be associated with RFI (Nafikov and Beitz, 2007; Karisa et al., 2014). Our research found that some key hub-genes are significantly enriched in fat metabolism-related pathways. These genes include *ECH*1, *EHHADH*, *CAT*, *ACSL*1, *ECI*2, *ABCD*2, *ACOX*1, and *PCK*1. Interestingly, these genes are both highly expressed in the high feed efficiency group in male/females. *ACSL*1 (acyl-CoA synthetase long-chain family member 1) plays an important role in the transportation and activation of fatty acids. A previous study in chickens showed that high expression of the *ACSL*1 gene could promote fat synthesis, while chickens with high feed efficiency tend to have more fat deposits (Neijat et al., 2017). The *PCK*1 gene is associated with obesity, insulin resistance, type II diabetes in mammals, and abdominal fat content (Beale et al., 2007; Rees et al., 2009; Millward et al., 2010). Previous research showed that fat deposition was positively correlated with *PCK*1 expression in birds.

Similarly, a prior study in chicken performance transcriptome sequencing also found PCK1 higher express in the LRFI group than HRFI; it was also speculated that LRFI chickens have more fatty deposits (Duan et al., 2013), which is consistent with the results in this study. A previous genome-wide association analysis related to pig feed efficiency found a significant correlation between the *ECI*2 (enoyl-CoA delta isomerase 2) gene and feed efficiency. Earlier studies in pigs showed that the expression level of the *ABCD*2 (ATP binding cassette subfamily D member 2) gene was downregulated in the high feed efficiency group, and this result was also verified in our study. In a meat duck liver transcriptome study, *ACOX*1 (acyl-CoA oxidase 1) expression was significantly negatively correlated with RFI, which was also consistent with our results. As for *ECH*1, *EHHADH*, and *CAT* genes, no relevant studies have shown that they have a relationship with RFI. It is challenging to predict chickens’ capability with low or high RFI to cause specific fat deposition changes. But our research confirmed that the regulatory relationship between feed efficiency and fat deposition needs to be further explored.

Interestingly, two key hub genes encode for Heat Shock Proteins (*HSP*s): H*SPA*2/H*SP90AA*1. Chickens are susceptible to heat stress due to their overall feather coverage and lack of sweat glands (Zhang C. et al., 2017). Numerous research has reported on the negative influence of heat stress on poultry production, such as decreased body antioxidant capacity and intestinal immunity and impaired intestinal morphology (Sahin et al., 2017; Song et al., 2018). Under HS conditions, chickens may spend more energy on maintenance and acclimation, which reduces the energy for growth and leads to a decrease in BWG (Mujahid et al., 2007). The activation of heat stress is energetically costly, and long-term stimulation can negatively impact feed efficiency. These genes involved in heat stress can be used as candidate markers related to feed efficiency. *HSPs* protein is highly conserved in all organisms and plays a crucial role in cellular stress response (Stetler et al., 2010). Many studies have demonstrated the link between heat stress and feed affection. Heat stress can lead to a decrease in animal feed intake and damage the intestine’s integrity and barrier function (Pearce et al., 2015). Studies have reported that when pigs undergo heat stress, the expression of HSP mRNA will increase. The abundance of various metabolic enzymes in the ileum will decrease, suggesting that the body’s metabolic process has changed (Pearce et al., 2015). Similar to our findings, a study explored the host transcriptome and microbiome interaction modulates of full-sibs broilers with divergent feed conversion ratio and identified that HSP90AA1 is a significantly differently expressed gene. In poultry production, strictly controlling the environment and avoiding heat stress will effectively improve feed efficiency.

We used the GSEA approach (Wang and Cairns, 2013) to compare biological differences in all gene expression patterns between the high-RFI and low-RFI groups. Based on the GO-based list, all higher expressed gene sets in the LRFI group were mainly divided into three categories. lipid metabolism, structure and function of small intestinal villi, and development of the nervous system. Some genes are enriched to the gene set involved in border brush, which is considered to be related to the intestine’s digestion and absorption function, including *MYO1D*, *MYO1E*, *MYO1A*, *USH1C*, and *EZR*. These genes in this pathway are highly expressed in the high feed efficiency group, which shows that some genes related to the intestinal structure may play an important role in regulating high feed efficiency chicken intestines with better absorption capacity. Biologists have long appreciated the intimate connection between morphology and function. Here, morphological adaptations at both the tissue and cellular level, allow the intestinal epithelium to make close and prolonged contact with luminal contents, promoting efficient uptake of available nutrients that may lead to the change of feed efficiency. The intestinal brush border is home to several class I myosins, with myosin-1a (myo1a) being the most abundant (McConnell et al., 2011). The expression of the myo1A gene has strong tissue specificity. Expression of myo1A is limited to the intestinal tract, where it localizes almost exclusively to the brush border (Skowron et al., 1998; Skowron and Mooseker, 1999). *MYO1A*, *MYO1D*, and *MYO1E* are considered to be key genes in the rapid differentiation of neonatal epithelial cells into mature intestinal epithelial cells (Tyska et al., 2005; Beale et al., 2007; Benesh et al., 2010). Previous studies have shown that a large deletion mutation in the human *USH1C* gene can cause severe gastrointestinal dysfunction (Bitner-Glindzicz et al., 2000; Hussain et al., 2004). Consistent with this, *USH1C* knockout mice, which were developed to model Type 1 Usher syndrome, display significant perturbations in intestinal brush border morphology (Crawley et al., 2014). Our results show the potential connection between chicken intestinal structure and function and feed efficiency. It is worth noting that in our GSEA enrichment analysis, some genes were enriched and related to nervous system development. A previous study integrated genome-wide co-association and gene expression found that some candidate genes directly or indirectly affect FE-related traits were mainly associated with immunity, nervous system, behavior, and energy metabolism (Ramayo-Caldas et al., 2019). The influence mechanism of nervous system development on feed efficiency needs further study.

To summarize, this work is the first to use RNA-seq in males and females to identify and annotate DEGs in the duodenum tissues of high- and low-FE native chickens. In male and females, we identified 1015 and 742 DEGs associated with RFI, respectively. Those genes were mainly enriched in the pathways of metabolism, oxidation reaction, and energy homeostasis. Moreover, we found that genes involved in fat metabolism and heat stress may affect feed efficiency through PPI network analysis. Through GSEA analysis, we found that many genes related to small intestinal villi structure and absorption function can be used as candidate genes to RFI, including *MYO1D*, *MYO1E*, *MYO1A*, *USH1C*, and *EZR*. Finally, the research shows that differences in gene expression patterns related to nervous system development may cause different feed efficiencies.

## Data Availability Statement

The sequencing data have been deposited in the NCBI SRA (http://www.ncbi.nlm.nih.gov/sra), BioProject accession PRJNA672913.

## Ethics Statement

The animal study was reviewed and approved by the Animal Ethics Committee of Guangxi University (GXU2018-058).

## Author Contributions

CX, JD, and ZY: data curation. CX, TS, ZY, and XY: formal analysis. XY: funding acquisition, project administration, and writing – review and editing. CX, JD, and LZ: investigation. CX and XY: methodology. JD and XY: resources. ZY and JD: supervision. TS and LZ: validation. CX and JD: writing – original draft. All authors contributed to the article and approved the submitted version.

## Conflict of Interest

The authors declare that the research was conducted in the absence of any commercial or financial relationships that could be construed as a potential conflict of interest.

## Footnotes

1 http://ftp.ensembl.org/pub/release-95/

2 http://string-db.org/cgi/input.pl
