# Supplementary material for: Transcriptome Analysis Identifies Candidate Genes and Signaling Pathways Associated With Feed Efficiency in Xiayan Chicken
Source: Front Genet. 2021 Mar 17;12:607719. doi: 10.3389/fgene.2021.607719 (PMC8010316; doi:10.3389/fgene.2021.607719)
Supplement: Supplementary file 1 [file Table_1.DOCX]

**Table-S1**

**List of qPCR Primers**

| **Gene primers** | **Direction** | **Primer sequence** |
| --- | --- | --- |
| SHH1 | Forward | AGCGATGAGGAGCGCAAAT |
| SHH1 | Reverse | GGGCAAATCACCTGCATGTTT |
| KMT2E | Forward | TGAAAATCCAGAGCCGCCTT |
| KMT2E | Reverse | GAGGAGGGCTTGCTTGCTTT |
| IQGAP2 | Forward | AACTCTGAGGAACCCAAACG |
| IQGAP2 | Reverse | GTCAGAGATTGTGCCGTTTACC |
| PPA2 | Forward | TGAGTGGCAGCCCTTTTTGT |
| PPA2 | Reverse | CCAGGAATCAACTTCTGGGCA |
| TRAFD1 | Forward | CATCACAGCAGCCACCATCT |
| TRAFD1 | Reverse | TGCACCCAGGAGAAACATCTTG |
| NPLOC4 | Forward | TGGCCACGTTCCTCAAGAAG |
| NPLOC4 | Reverse | TCGCCATGCTTGATTTTCAGT |
