# Supplementary material for: Transcriptome Analysis Identifies Candidate Genes and Signaling Pathways Associated With Feed Efficiency in Xiayan Chicken
Source: Front Genet. 2021 Mar 17;12:607719. doi: 10.3389/fgene.2021.607719 (PMC8010316; doi:10.3389/fgene.2021.607719)
Supplement: Supplementary file 2 [file Table_2.DOCX]

**Table S2:** Summary of sequencing reads mapping to the reference genome and quality parameters

| **sample** | **raw reads** | **clean reads** | **Total mapped** | **Uniquely mapped** | **Q20(%)** | **Q30(%)** | **GC content(%)** |
| --- | --- | --- | --- | --- | --- | --- | --- |
| MH3 | 80901786 | 78143232 | 89.45% | 81.82% | 95.88 | 90.05 | 48.45 |
| FH1 | 83037376 | 80492210 | 89.26% | 81.02% | 96.18 | 90.65 | 48.46 |
| ML2 | 74748800 | 55314196 | 88.63% | 81.11% | 97.04 | 92.89 | 48.86 |
| MH2 | 83892104 | 81428162 | 90.39% | 81.68% | 96.36 | 91.02 | 48.87 |
| MH1 | 80241568 | 77695788 | 89.70% | 81.22% | 96.05 | 90.38 | 48.91 |
| ML3 | 1.05E+08 | 102421430 | 91.79% | 81.42% | 97.51 | 93.7 | 48.94 |
| FL1 | 52314366 | 50275148 | 85.41% | 77.73% | 97.26 | 93.35 | 49.27 |
| FH3 | 60956180 | 59497818 | 87.11% | 79.40% | 96.08 | 90.74 | 49.34 |
| FH2 | 70099814 | 68527196 | 86.68% | 79.55% | 96.29 | 91.13 | 49.97 |
| FL2 | 67533240 | 64866318 | 81.92% | 74.43% | 94.63 | 87.97 | 50.34 |

Notes: Raw reads: total number of pair-end reads in the raw data; Clean reads: total number of pair-end reads in the clean data; Q20 (%): the percentage of the Q20 base; Q30 (%): the percentage of the Q30 base; Total Maped(%):the percentage of the clean reads map to the Reference genome; Unique Maped(%):the percentage of the clean reads map to the Reference genome unique position; GC content (%): percentage of G and C bases in the clean data.
